# Healthcare workers' knowledge and attitudes regarding artificial intelligence adoption in healthcare: A cross-sectional study

**DOI:** 10.1016/j.heliyon.2024.e40775

**Published:** 2024-11-29

**Authors:** Moustaq Karim Khan Rony, Khadiza Akter, Latifun Nesa, Md Tawhidul Islam, Fateha Tuj Johra, Fazila Akter, Muhammad Join Uddin, Jeni Begum, Md. Abdun Noor, Sumon Ahmad, Sabren Mukta Tanha, Most. Tahmina Khatun, Shuvashish Das Bala, Mst. Rina Parvin

**Affiliations:** a Master of Public Health, Bangladesh Open University, Gazipur, Bangladesh; b Master of Public Health, Daffodil International University, Dhaka, Bangladesh; c Master’s of Child Health Nursing, National Institute of Advanced Nursing Education and Research Mugda, Dhaka, Bangladesh; d Lecturer, North East Nursing College, Sylhet, Bangladesh; e Masters in Disaster Management, University of Dhaka, Dhaka, Bangladesh; f Dhaka Nursing College, Affiliated with the University of Dhaka, Bangladesh; g Master of Public Health, RTM Al-Kabir Technical University, Sylhet, Bangladesh; h Master of Public Health, Leading University, Sylhet, Bangladesh; i School of Medical Sciences, Shahjalal University of Science and Technology, Sylhet, Bangladesh; j Rajshahi Medical College Hospital, Rajshahi, Bangladesh; k Associate Professor, College of Nursing, International University of Business Agriculture and Technology, Dhaka, Bangladesh; l Major at Bangladesh Army (AFNS Officer), Combined Military Hospital, Dhaka, Bangladesh; m Department of Health and Functioning, Western Norway University of Applied Sciences, Norway

**Keywords:** Artificial intelligence, Healthcare workers, Technology, Medical practice, Knowledge, Attitude

## Abstract

**Background:**

The convergence of healthcare and artificial intelligence (AI) introduces a transformative era in medical practice. However, the knowledge and attitudes of healthcare workers concerning the adoption of artificial intelligence in healthcare are currently unknown.

**Aims:**

The primary objective was to investigate the knowledge and attitudes of healthcare professionals in Dhaka city, Bangladesh, regarding the adoption of AI in healthcare.

**Methods:**

A cross-sectional research design was employed, incorporating a dual-method approach to select participants using randomness and convenience sampling techniques. Validity was ensured through a literature review, content validity, and reliability assessment (Cronbach's alpha = 0.85), and exploratory factor analysis identified robust underlying factors. Data analysis involved descriptive and inferential statistics, including Fisher's exact tests, multivariate logistic regression, and Pearson correlation analysis, conducted using STATA software, providing a comprehensive understanding of healthcare workers' AI adoption in healthcare.

**Results:**

This study revealed that age was a significant factor, with individuals aged 18–25 and 26–35 having higher odds of good knowledge and positive attitudes (AOR 1.56, 95 % CI 1.12–2.43; AOR 1.42, 95 % CI 0.98–2.34). Physicians (AOR 1.08, 95 % CI 0.78–1.89), hospital workers (AOR 1.29, 95 % CI 0.92–2.09), and full-time employees (AOR 1.45, 95 % CI 1.12–2.34) exhibited higher odds. Attending AI conferences (AOR 1.27, 95 % CI 0.92–2.23) and learning through research articles/journals (AOR 1.31, 95 % CI 0.98–2.09) were positively associated with good knowledge and positive attitudes. This research also emphasized the strong correlations between knowledge and positive attitudes (r = 0.89, P < 0.001), as well as negative attitudes with poor knowledge (r = 0.65, P < 0.001).

**Conclusions:**

The study highlights the critical need for targeted educational interventions to bridge the knowledge gaps among healthcare professionals regarding AI adoption. The findings reveal that younger healthcare workers, those in full-time employment, and individuals with exposure to AI through conferences or research are more likely to possess good knowledge and hold positive attitudes towards AI integration. These results suggest that policies and training programs must be tailored to address specific demographic differences, ensuring that all groups are equipped to engage with AI technologies. Moreover, the study emphasizes the importance of continuous professional development, which could foster a workforce capable of harnessing AI's potential to improve patient outcomes and healthcare efficiency.

## Highlights

• 62.18% recognize AI's role in patient outcomes; 51.51% see its value in early disease detection and diagnosis.
• 50.58% believe AI can enhance healthcare; 57.54% are comfortable using AI tools in daily tasks.
• Younger (18–35) and male participants show better knowledge and positive attitudes toward AI in healthcare.
• A strong positive correlation (r = 0.89) exists between knowledge and positive attitudes toward AI in healthcare.
• 39.26% show good AI knowledge, while 73.06% have a positive attitude toward its adoption in healthcare.

## 1 Introduction

The dynamic intersection of healthcare and technology has ushered in a new era marked by transformative advancements, with artificial intelligence (AI) emerging as a pivotal force reshaping the landscape of medical practice [1,2]. In this era of unprecedented innovation, the amalgamation of healthcare and technology has led to groundbreaking developments that hold the promise of revolutionizing patient care [3,4]. Healthcare and technology, once distinct domains, have become increasingly intertwined, with technology acting as a catalyst for transformative changes in the delivery of healthcare services [[5], [6], [7]]. The implementation of electronic health records (EHRs), telemedicine, and advanced diagnostic tools has significantly improved healthcare delivery efficiency while laying the foundation for the seamless integration of AI into modern medical practice [8,9]. As the boundaries between these traditionally separate realms blur, the healthcare sector stands at the forefront of a technological revolution, with AI positioned as a key player in shaping the future of patient care [10,11].

The evolution of machine learning algorithms, the availability of vast datasets for training AI models, and advancements in computational power have collectively contributed to the rise of AI applications in healthcare [12,13]. Machine learning, a subset of AI, has proven particularly influential, enabling computers to analyze and learn from data patterns, thereby enhancing their ability to make predictions and decisions [14,15]. The utilization of AI in medical imaging, disease diagnosis, and treatment optimization has showcased its potential to augment the capabilities of healthcare professionals and improve patient outcomes [16,17]. These key developments underscore the transformative power of AI, marking a paradigm shift in the way healthcare is conceptualized and delivered.

The transformative role of artificial intelligence in healthcare is profound. AI presents numerous opportunities, ranging from creating personalized treatment plans tailored to individual patient data to enhancing healthcare processes [18,19]. With the ability to analyze vast datasets at unprecedented speeds, AI has the potential to revolutionize diagnostic accuracy, treatment efficacy, and even predict and prevent diseases [20]. The advent of AI-driven technologies holds the promise of ushering in a new era of precision medicine, where interventions are tailored to individual patient characteristics, leading to more effective and targeted healthcare outcomes [21,22]. Moreover, the integration of AI has the potential to alleviate the burden on healthcare systems, streamlining workflows and allowing healthcare professionals to focus on high-value, patient-centric care [23,24].

As the healthcare industry undergoes this paradigm shift, the role of healthcare workers in the adoption of AI becomes paramount. Healthcare professionals, ranging from doctors and nurses to administrative staff, are at the forefront of patient care, making their perspectives and acceptance of AI technologies crucial for successful integration [25,26]. The effectiveness of AI in healthcare is contingent on the collaboration between these professionals and the technologies they employ [27,28]. Grasping the role and perspectives of healthcare workers in the adoption of AI is essential for crafting strategies that foster a harmonious partnership between human expertise and technological innovation [29,30].

In light of AI permeating various facets of medical practice, it is imperative to gauge healthcare workers' familiarity with AI concepts and their perceptions of its integration into daily routines [[31], [32], [33]]. This study addresses a significant gap in understanding the knowledge and attitudes of healthcare professionals regarding AI adoption in healthcare settings. While AI technologies are rapidly evolving, little is known about the perspectives of those who directly engage with these technologies. By focusing on healthcare workers' familiarity with AI concepts and their attitudes toward its implementation, this research aims to provide insights into the current state of their knowledge about AI, revealing the depth of their understanding of its applications and assessing their attitudes towards its adoption.

The findings of this study are vital for developing targeted educational programs and interventions that cater to specific needs and concerns, facilitating a smoother transition to an AI-enabled healthcare environment. Additionally, this research is novel in its exploration of demographic factors influencing AI adoption, an area that has been underexplored in the existing literature. This comprehensive approach not only enriches the discourse surrounding AI in healthcare but also empowers stakeholders with the knowledge necessary to foster a more informed and collaborative healthcare workforce, ultimately enhancing patient care and outcomes.

## 2 Methodology

### 2.1 Study design

Between August 2023 and February 2024, we utilized a cross-sectional research design in Dhaka city, Bangladesh, aiming to acquire a comprehensive understanding of healthcare workers' perspectives on the integration of artificial intelligence (AI) in healthcare. The adoption of a cross-sectional approach facilitated the collection of data at a specific moment, providing a contemporaneous portrayal of healthcare professionals' attitudes and knowledge regarding the incorporation of AI into healthcare practices.

#### 2.1.1 Population and sampling

This study aimed to engage a varied group of healthcare professionals, encompassing doctors, nurses, therapists, technicians, and administrative staff. The research adopted a stratified random sampling approach [34], stratifying the city based on pertinent characteristics like geographic location and healthcare facility type. From each stratum, representative samples were randomly selected, ensuring a diverse and comprehensive array of healthcare settings. For participant selection within these settings, a dual methodology involving both random and convenience sampling techniques was employed [35]. Random sampling ensured equitable representation of individuals within healthcare settings, while convenience sampling facilitated practical considerations, such as ease of access to specific facilities [36]. The deliberate equilibrium between randomness and representation in both healthcare settings and participant selection aimed to yield reliable survey outcomes. This approach facilitated the inclusion of participants from various healthcare environments, encompassing hospitals, clinics, diagnostic centers, and nursing homes. The meticulous sampling strategy enhances the study's credibility, ensuring a robust and inclusive examination of healthcare professionals' knowledge and attitudes regarding the adoption of artificial intelligence in healthcare across Dhaka city, Bangladesh.

### 2.2 Data collection

#### 2.2.1 Inclusion criteria

Participants in this study were required to meet specific inclusion criteria. They must be actively employed in healthcare settings and have a minimum of six months of direct patient care experience. This requirement ensures that participants have firsthand insights into clinical practices, allowing them to provide valuable perspectives on the integration of artificial intelligence in real-world healthcare contexts. Furthermore, participants needed to have completed at least three years of diploma courses or degrees, emphasizing the significance of educational background and professional development in shaping their attitudes and knowledge regarding AI in healthcare. This dual-criteria approach was designed to assemble a well-informed and diverse group of participants, thereby enhancing the study's depth and relevance.

##### 2.2.1.1 Exclusion criteria

In contrast, individuals who did not meet certain criteria were excluded from the study. Those not actively employed in healthcare settings, who had less than six months of direct patient care experience, or who lacked the minimum educational qualifications were not eligible to participate. These exclusion criteria were put in place to ensure that all participants possessed the necessary experience and knowledge to contribute meaningfully to the research.

#### 2.2.2 Survey instrument

A meticulously crafted questionnaire served as the primary data collection tool. This questionnaire was adopted from an extensive literature review [[37], [38], [39], [40]]. Sections of the questionnaire covered demographics, AI knowledge, and attitudes towards AI adoption. To facilitate quantitative data collection, the questionnaire included a mix of closed-ended and Likert scale questions.

#### 2.2.3 Pilot testing

In the pursuit of refining the questionnaire, a pilot test was conducted with a small group [41] of healthcare workers (n = 41). Valuable feedback obtained during this phase played a crucial role in informing necessary refinements, ultimately enhancing the clarity, relevance, and reliability of the questionnaire. This iterative process not only ensured the instrument's validity but also bolstered its effectiveness in capturing the intended data. The insights gained from the pilot testing phase contributed to the overall robustness of the survey instrument, setting the stage for a more comprehensive and insightful data collection process among healthcare professionals.

### 2.3 Data collection procedure

**Fig. 1:**
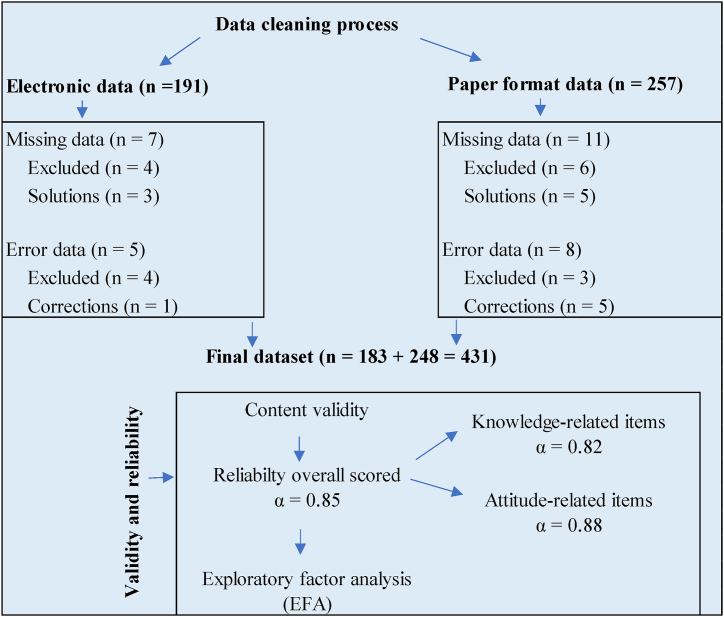
Flow chart for questionnaire items: validity, reliability, and data cleaning process.

We collected data using self-administered surveys, distributed either electronically or in paper format according to participants' preferences (Fig. 1). Prior to participation, participants were extensively briefed on the study's objectives, and their informed consent was obtained. Emphasizing the significance of candid responses, the study prioritized confidentiality and anonymity to create a comfortable environment. This thorough data collection process aimed to both adhere to ethical standards and encourage an authentic and uninhibited sharing of healthcare workers' knowledge, and attitudes concerning the integration of artificial intelligence in healthcare.

### 2.4 Variables and measures

#### 2.4.1 Dependent and independent variables

This study focused on evaluating two primary dependent variables: healthcare workers' knowledge about AI and their attitudes towards the adoption of AI in healthcare. Independent variables included a range of demographic factors such as age, gender, educational background, years of working experience, job role, work setting, employment status, and attendance at any national or international conferences.

#### 2.4.2 Measurement scales

Knowledge about AI: A set of 14 multiple-choice questions [39,40] was used to assess participants' comprehension of fundamental AI concepts, their applications in healthcare, and associated benefits and risks. Scores ranging from 0 to 7 indicated poor knowledge, while scores from 8 to 14 indicated good knowledge regarding the adaptation of AI in healthcare.

Attitudes towards AI adoption: Participants expressed their opinions on various aspects using a 5-point Likert scale [37,38]. This included AI's influence on healthcare efficiency, diagnostic accuracy, treatment planning, concerns about potential job displacement, impact on daily healthcare tasks, and overall quality of care. Scores ranging from 0 to 8 indicated a negative attitude, while scores from 9 to 18 indicated a positive attitude towards AI adoption in healthcare. This comprehensive approach to variable measurement ensures a nuanced understanding of both knowledge levels and attitudes among healthcare workers, contributing to a more thorough analysis of AI integration in the healthcare sector.

### 2.5 Sample size calculations

The determination of an optimal sample size is crucial to ensure the reliability and validity of findings. The sample size calculation was guided by the formula [42]:

$$i.\;n=\frac{Z^{2}\;X\;P\;X\;\left(1-P\right)}{E^{2}}$$

Where,

n = required sample size.

Z = Z-score (1.96 for a 95 % confidence level)

*p* = estimated proportion (0.5 for maximum variability)

*E* = margin of error (0.05)

Substituting the values, we have:

$$ii.\;n=\frac{{\left(1.96\right)}^{2}\;X\;0.5X\;\left(1-0.5\right)}{{\left(0.05\right)}^{2}}$$

=384.16≈384

According to this calculation, 384 healthcare workers need to be included in the study to achieve a 95 % confidence level with a 5 % margin of error. Adhering to a carefully calculated sample size ensures the study's ability to detect significant patterns or trends within the population of healthcare workers, thereby enhancing the overall statistical power and generalizability of findings. This meticulous approach is particularly critical when investigating nuanced topics like the integration of artificial intelligence in healthcare.

### 2.6 Data analysis

In this study, descriptive statistics, including frequencies and percentages, served as foundational tools for succinctly summarizing participant demographics and key variables. This provided an initial overview, offering valuable insights into the characteristics of the study's participants. Moving beyond descriptive statistics, this research incorporated inferential statistics to unravel more intricate relationships within the data. Specifically, Fisher's exact tests were utilized [43] to explore associations between demographic variables and healthcare workers' knowledge and attitudes towards the adoption of artificial intelligence (AI). This statistical method aids in discerning whether there are significant associations between categorical variables, shedding light on potential factors influencing healthcare professionals' perspectives on AI integration.

Furthermore, multivariate logistic regression was employed to delve deeper into the complex interplay of various independent variables [44]. This sophisticated statistical technique allows the exploration of relationships between multiple independent variables and the binary outcome variables of knowledge and attitudes towards AI adoption. By considering the simultaneous impact of several variables, multivariate logistic regression offers nuanced insights into the factors influencing healthcare workers' knowledge and attitudes in a more comprehensive manner. Additionally, Pearson correlation analysis was employed to assess the strength and direction of linear relationships between continuous variables [44]. This analytical tool aids in uncovering potential correlations between variables, providing valuable insights into the linear associations that may exist among different factors influencing healthcare workers' perceptions of AI adoption.

### 2.7 Validity and reliability

To assess the validity of the research instrument, the study conducted an extensive literature review on artificial intelligence adoption in healthcare. The survey questions were meticulously crafted to align with established knowledge and attitude constructs relevant to the subject matter. This thorough approach significantly contributed to achieving a high level of content validity. Regarding reliability, Cronbach's coefficient alpha was computed for the survey instrument, yielding a value of α = 0.85 for the overall instrument (Fig. 1). For knowledge-related items, the Cronbach's alpha was 0.82, signifying strong internal consistency among questions related to knowledge constructs. For attitude-related items, the Cronbach's alpha was 0.88, indicating robust internal consistency among questions related to attitude constructs. Both values surpass the widely accepted threshold of 0.70, underscoring the dependability and consistency of the collected data for both knowledge and attitude dimensions. The research additionally employed exploratory factor analysis (EFA) to evaluate construct validity. The analysis identified three underlying factors that contribute to healthcare workers' knowledge and attitudes regarding AI adoption, featuring eigenvalues exceeding 1.0 and factor loadings greater than 0.60. Together, these factors collectively accounted for 78 % of the variance in the data, pointing to a robust factor structure.

### 2.8 Ethical considerations

Prior to initiating the study, the investigators diligently secured approval from the ethics review board of Mahbubur Rahman Memorial Hospital & Nursing Institute (approval number: MRMHNI/IRB/HR-20230513) on May 5, 2023, affirming their commitment to ethical guidelines and the protection of the well-being of participating healthcare workers. All participants provided informed consent, emphasizing the study's objectives, potential risks, and the voluntary nature of their involvement. Stringent confidentiality measures were implemented to safeguard participant privacy, ensuring that responses remained anonymized and securely stored. To address potential psychological or emotional impacts on participants, the survey instrument was thoughtfully designed with sensitivity to the nature of the questions. Participants were reassured of their right to withdraw from the study at any point without facing consequences. The dissemination of research findings adhered to ethical principles, with a focus on transparent reporting to avoid sensationalism or distortion of results. These ethical considerations underscore the researchers' dedication to upholding the rights and well-being of healthcare workers, thereby maintaining the integrity of the research process and its outcomes.

## 3 Results

### 3.1 Demographic profile

In Table 1, detailing the demographic profile of 431 participants, several key variables stand out. Notably, a significant portion of respondents fell within the age range of 18–25 years (40.37 %) and 26–35 years (39.68 %). Gender distribution is relatively balanced, with the highest responses from females (58.93 %) and males (33.41 %). Diverse educational backgrounds are evident, with the top two categories being participants with a diploma course (35.73 %) and those with a bachelor's degree/MBBS (30.86 %). The majority of respondents had 1–5 years of healthcare experience (39.21 %), and nurses (67.52 %) dominate the top job roles, followed by physicians (18.79 %). Hospitals are the primary work setting (67.52 %), and full-time employment is prevalent (70.3 %). A notable 26.68 % attended national or international AI conferences in healthcare. Facebook/YouTube (32.48 %) and TV news (34.34 %) emerged as the leading sources for AI adoption knowledge, indicating the influence of digital and traditional media in shaping participants' awareness of AI in healthcare.

**Table 1:** Presents the demographic profile of the 431 participants.

**Variables**	**N**	**%**
Age		
18–25 years	174	40.37
26–35 years	171	39.68
36–45 years	45	10.44
46–55 years	31	7.19
≥56 years	10	2.32
**Gender**		
Male	144	33.41
Female	254	58.93
Non-binary	13	3.02
Prefer not to say	20	4.64
**Educational background**		
Diploma course	154	35.73
Bachelor's degree/MBBS	133	30.86
Master's degree	107	24.83
Doctorate	19	4.41
Other	18	4.18
**Years of experience in healthcare**		
Less than 1 year	116	26.91
1–5 years	169	39.21
6–10 years	93	21.58
11–15 years	33	7.66
16 years and above	20	4.64
**Job role**		
Physician	81	18.79
Nurse	291	67.52
Therapist/Medical technologist	13	3.02
Administrator/Manager	5	1.16
Other	41	9.51
**Work setting**		
Hospital	291	67.52
Clinic/Outpatient facility	18	4.18
Diagnostic center	35	8.12
Nursing home/Long-term care	6	1.39
Other	81	18.79
**Employment status**		
Full-time	303	70.3
Part-time	18	4.18
Contract/Temporary	47	10.9
Other	63	14.62
**Did you attend any national or international conference regarding artificial intelligence adoption in healthcare?**		
Yes	115	26.68
No	316	73.32
**How did you learn about artificial intelligence adoption in healthcare?**		
By attending a conference	19	4.41
Facebook/YouTube	140	32.48
Online news portal	48	11.14
Research articles/Journal websites	43	9.98
TV news	148	34.34
Other	33	7.66

### 3.2 Participants' knowledge

Table 2 presents an overview of participants' knowledge regarding the adoption of artificial intelligence (AI) in healthcare. A majority recognized AI's potential to improve patient outcomes (62.18 %) and its role in early disease detection and diagnosis (51.51 %). Familiarity with AI's contributions to personalized medicine and treatment plans was reported at 42.46 %, while awareness of ethical considerations was noted by 42.00 %. Participants demonstrated knowledge of AI's efficiency in administrative tasks (52.44 %) and its impact on medical imaging accuracy and speed (56.15 %). Additionally, 49.65 % were aware of the importance of security and privacy measures for patient data in AI applications, and 51.28 % understood the need for training healthcare workers to integrate AI tools effectively. Awareness of AI's potential impact on job roles was reported by 52.20 %, with 56.38 % recognizing its contribution to predictive analytics. Participants also acknowledged how AI supports routine task automation (53.13 %) and its cost-effectiveness in healthcare (46.17 %).

**Table 2:** Illustrates the distribution of knowledge and attitudes regarding the adoption of Artificial Intelligence in Healthcare among a sample size of 431 participants.

**Questions**	**Correct answer N (%)**
**Knowledge**	
How can artificial intelligence (AI) improve patient outcomes in healthcare settings?	268 (62.18)
What role can AI play in early disease detection and diagnosis?	222 (51.51)
How does AI contribute to personalized medicine and treatment plans?	183 (42.46)
What are the potential challenges and ethical considerations associated with AI adoption in healthcare?	181 (42.00)
How can AI enhance the efficiency of administrative tasks within healthcare organizations?	226 (52.44)
In what ways can AI be utilized to improve the accuracy and speed of medical imaging analysis?	242 (56.15)
What measures should be in place to ensure the security and privacy of patient data in AI applications?	214 (49.65)
How can healthcare workers be trained to effectively integrate AI tools into their daily workflows?	221 (51.28)
What impact might AI have on the job roles and responsibilities of healthcare professionals?	225 (52.20)
How does AI contribute to predictive analytics for disease prevention and patient management?	243 (56.38)
What are the key factors influencing the successful implementation of AI in healthcare?	202 (46.87)
How can AI support the automation of routine tasks, allowing healthcare workers to focus on more complex and critical aspects of patient care?	229 (53.13)
What evidence exists regarding the cost-effectiveness of AI adoption in healthcare?	199 (46.17)
How can healthcare organizations ensure that AI technologies are accessible and beneficial to diverse patient populations?	188 (43.62)
**Attitude**	**Maximum response**

I believe that artificial intelligence (AI) has the potential to significantly improve healthcare services.	Agree 218 (50.58)
I feel confident in my understanding of how artificial intelligence functions in healthcare settings.	Confident 209 (48.49)
I am open to incorporating artificial intelligence tools and technologies into my daily healthcare practice.	Open 225 (52.20)
I believe that artificial intelligence could enhance diagnostic accuracy and treatment planning in healthcare.	Strongly agree 175 (40.60)
I am concerned about the potential job displacement of healthcare workers due to increased use of artificial intelligence.	Concerned 200 (46.40)
I think proper training and education on artificial intelligence are essential for healthcare professionals.	Strongly agree 214 (49.65)
I believe that the integration of artificial intelligence in healthcare will lead to more efficient and cost-effective patient care.	Agree 185 (42.92)
I feel comfortable using artificial intelligence tools in my daily healthcare tasks.	Comfortable 248 (57.54)
I perceive that my colleagues are generally supportive of the adoption of artificial intelligence in healthcare.	Agree 220 (51.04)

### 3.3 Participants' attitude

Table 2 provides a comprehensive overview of the attitudes held by 431 participants regarding the adoption of artificial intelligence (AI) in healthcare. Notably, 50.58 % agreed that AI has significant potential to enhance healthcare services. Confidence in understanding AI's functions was reported by 48.49 %, while 52.20 % expressed openness to incorporating AI tools into their daily practices. Strong agreement regarding AI's ability to improve diagnostic accuracy and treatment planning was voiced by 40.60 %. Concerns about job displacement due to AI were acknowledged by 46.40 %, and the need for proper training and education on AI was emphasized by 49.65 %. A positive outlook on AI integration leading to more efficient and cost-effective care was shared by 42.92 %, with 57.54 % feeling comfortable using AI tools in their daily tasks. Additionally, 51.04 % perceived support among colleagues f or AI adoption, reflecting a mix of optimism, openness, and concerns regarding AI in healthcare.

### 3.4 Distribution of participants' knowledge and positive attitudes in relation to independent variables

Table 3 highlights significant findings related to the distribution of good knowledge and positive attitudes toward artificial intelligence (AI) adoption in healthcare. Age differences were notable, with individuals aged 18–25 and 26–35 demonstrating higher levels of good knowledge (p < 0.001 and p = 0.021) and positive attitudes (p < 0.001). Males exhibited superior knowledge and attitudes compared to other genders (p < 0.001). Educational background significantly influenced results, as individuals with Bachelor's, Master's, and Doctorate degrees had higher knowledge and positive attitudes (p < 0.001). Years of experience also mattered, with those having less than 1 year or 1–5 years of experience showing better outcomes (p < 0.001 and p = 0.018). Job roles, particularly physicians and nurses, were associated with higher knowledge and attitudes (p < 0.001). Additionally, full-time employees, those attending conferences, and individuals learning about AI through research articles/journal websites exhibited significantly higher levels of knowledge and positive attitudes (p < 0.001).

**Table 3:** Displays the distribution of participants' good knowledge and positive attitudes based on their demographic profile.

**Variables (n = 431)**	**Good knowledge (n = 169)**		**Positive attitude (n = 315)**	
	**N (%)**	***P∗***	**N (%)**	***P∗***
Age				
18–25 years (n = 174)	97 (55.75)	***<0.001***	157 (90.23)	***<0.001***
26–35 years (n = 171)	49 (28.65)	***0.021***	135 (78.95)	***<0.001***
36–45 years (n = 45)	12 (26.67)	0.309	15 (33.33)	0.103
46–55 years (n = 31)	8 (25.8)	0.072	6 (19.35)	0.651
≥56 years (n = 10)	3 (30)	0.095	2 (20)	0.124
**Gender**				
Male (n = 144)	108 (75)	***<0.001***	123 (85.42)	***<0.001***
Female (n = 254)	54 (21.26)	0.137	181 (71.26)	***<0.001***
Non-binary (n = 13)	3 (23.08)	0.142	4 (30.77)	0.195
Prefer not to say (n = 20)	4 (20)	0.159	7 (35)	0.427
**Educational background**				
Diploma course (n = 154)	16 (10.39)	0.198	81 (52.60)	0.204
Bachelor's degree/MBBS (n = 133)	71 (53.38)	***0.013***	114 (85.71)	***<0.001***
Master's degree (n = 107)	64 (59.81)	***<0.001***	97 (90.65)	***<0.001***
Doctorate (n = 19)	15 (78.95)	***<0.001***	18 (94.74)	***<0.001***
Other (n = 18)	3 (16.67)	0.219	5 (27.78)	0.224
**Years of experience in healthcare**				
Less than 1 year (n = 116)	78 (67.24)	***<0.001***	109 (93.97)	***<0.001***
1–5 years (n = 169)	61 (36.09)	***0.018***	147 (86.98)	***<0.001***
6–10 years (n = 93)	23 (24.73)	0.627	52 (55.91)	0.522
11–15 years (n = 33)	4 (12.12)	0.198	5 (15.15)	0.175
16 years and above (n = 20)	3 (15)	0.572	2 (10)	0.461
**Job role**				
Physician (n = 81)	63 (77.78)	***<0.001***	72 (88.89)	***<0.001***
Nurse (n = 291)	97 (33.33)	***<0.001***	226 (77.64)	***<0.001***
Therapist/Medical technologist (n = 13)	2 (15.38)	0.596	5 (38.46)	0.662
Administrator/Manager (n = 5)	1 (20)	0.374	2 (40)	0.213
Other (n = 41)	6 (14.63)	0.318	10 (40.39)	0.431
**Work setting**				
Hospital (n = 291)	126 (43.30)	***<0.001***	245 (84.16)	***<0.001***
Clinic/Outpatient facility (n = 18)	5 (27.78)	0.345	8 (44.44)	0.577
Diagnostic center (n = 35)	15 (42.86)	***<0.001***	29 (82.86)	***<0.001***
Nursing home/Long-term care (n = 6)	2 (33.33)	0.385	1 (16.67)	0.188
Other (n = 81)	21 (25.93)	0.129	32 (39.51)	0.512
**Employment status**				
Full-time (n = 303)	145 (47.85)	***<0.001***	269 (88.78)	***<0.001***
Part-time (n = 18)	3 (16.67)	0.409	4 (22.22)	0.18
Contract/Temporary (n = 47)	9 (19.15)	0.291	17 (36.17)	0.389
Other (n = 63)	12 (19.05)	0.424	25 (39.68)	0.551
**Did you attend any national or international conference regarding artificial intelligence adoption in healthcare?**				
Yes (n = 115)	91 (79.13)	***<0.001***	112 (97.39)	***<0.001***
No (n = 316)	78 (24.68)	0.292	203 (64.24)	0.451
**How did you know about the adoption of artificial intelligence in healthcare?**				
By attending a conference (n = 19)	14 (73.68)	***<0.001***	17 (89.47)	***<0.001***
Facebook/YouTube (n = 140)	44 (31.43)	0.362	94 (67.14)	0.238
Online news portal (n = 48)	32 (66.67)	0.216	42 (87.50)	0.519
Research articles/Journal websites (n = 43)	38 (88.37)	***<0.001***	43 (100)	***<0.001***
TV news (n = 148)	36 (24.32)	0.498	106 (71.62)	0.573
Other (n = 33)	5 (15.15)	0.368	13 (39.39)	0.126

Values in bold represent significant results. ∗ p-values were from Chi-square test.

### 3.5 Association between dependent and independent variables

Table 4 presents the results of a multivariable logistic regression analysis examining the associations between healthcare workers' demographic characteristics and their knowledge and attitudes toward artificial intelligence (AI) adoption in healthcare. Notably, age significantly influenced both knowledge and positive attitudes. Healthcare workers aged 18–25 demonstrated a higher likelihood of possessing good knowledge (AOR 1.56, 95 % CI 1.12–2.43) and a positive attitude (AOR 1.42, 95 % CI 0.98–2.34). Similarly, those aged 26–35 had increased odds of good knowledge (AOR 1.27, 95 % CI 0.86–2.09) and a positive attitude (AOR 1.14, 95 % CI 0.78–1.97).

**Table 4:** Presents the results of a multivariable logistic regression analysis, examining the associations between the demographic characteristics of healthcare workers and their good knowledge and positive attitude toward the adoption of artificial intelligence in healthcare (n = 431).

**Variables (n = 431)**	**Good knowledge (n = 169)**		**Positive attitude (n = 315)**	
	**AOR (95%CI)**		**AOR (95%CI)**	
Age rowhead				
≥56 years	Reference		Reference	
18–25 years	**1.56 (1.12**–**2.43)**		**1.42 (0.98**–**2.34)**	
26–35 years	**1.27 (0.86**–**2.09)**		**1.14 (0.78**–**1.97)**	
36–45 years	1.08 (0.75–1.92)		0.88 (0.62–1.59)	
46–55 years	0.75 (0.42–1.33)		0.65 (0.38–1.15)	
**Gender** rowhead				
Prefer not to say	Reference		Reference	
Male	**1.35 (0.92**–**2.14)**		**1.18 (0.78**–**2.07)**	
Female	1.23 (0.84–2.09)		**1.09 (0.74**–**1.98)**	
Non-binary	0.94 (0.62–1.72)		0.97 (0.62–1.81)	
**Educational background** rowhead				
Other	Reference		Reference	
Diploma course	0.62 (0.38–1.09)		0.88 (0.56–1.69)	
Bachelor's degree/MBBS	**0.82 (0.49**–**1.54)**		**1.03 (0.72**–**1.89)**	
Master's degree	**1.11 (0.78**–**1.89)**		**1.45 (1.02**–**2.37)**	
Doctorate	**1.67 (1.21**–**2.49)**		**1.53 (1.12**–**2.34)**	
**Years of experience in healthcare** rowhead				
16 years and above	Reference		Reference	
Less than 1 year		**1.28 (0.92**–**2.09)**		**1.42 (0.98**–**2.34)**
1–5 years		**1.14 (0.86**–**1.97)**		**1.19 (0.82**–**2.01)**
6–10 years		0.68 (0.42–1.17)		0.85 (0.58–1.57)
11–15 years	0.91 (0.58–1.69)		0.93 (0.62–1.69)	
**Job role** rowhead				
Other	Reference		Reference	
Physician	**1.08 (0.78**–**1.89)**		**1.16 (0.82**–**1.97)**	
Nurse	**0.96 (0.68**–**1.81)**		**0.89 (0.62**–**1.54)**	
Therapist/Medical technologist	1.24 (0.92–2.09)		1.37 (1.02–2.23)	
Administrator/Manager	0.81 (0.52–1.47)		1.01 (0.74–1.69)	
**Work setting** rowhead				
Other	Reference		Reference	
Hospital	**1.29 (0.92**–**2.09)**		**1.45 (1.12**–**2.34)**	
Clinic/Outpatient facility	0.67 (0.42–1.15)		0.92 (0.68–1.69)	
Diagnostic center	**1.12 (0.86**–**1.69)**		**1.04 (0.78**–**1.89)**	
Nursing home/Long-term care	0.88 (0.62–1.54)		0.99 (0.62–1.81)	
**Employment status** rowhead				
Other	Reference		Reference	
Full-time	**1.45 (1.12**–**2.34)**		**1.33 (0.98**–**2.09)**	
Part-time	0.71 (0.46–1.29)		0.76 (0.52–1.41)	
Contract/Temporary	1.21 (0.86–2.01)		1.09 (0.82–1.89)	
**Did you attend any national or international conference regarding artificial intelligence adoption in healthcare?** rowhead				
No	Reference		Reference	
Yes	**1.27 (0.92**–**2.23)**		**1.47 (1.12**–**2.34)**	
**How did you know about the adoption of artificial intelligence in healthcare?** rowhead				
Other	Reference		Reference	
By attending a conference	**1.47 (1.12**–**2.34)**		**1.22 (0.86**–**1.97)**	
Facebook/YouTube	0.72 (0.46–1.29)		0.81 (0.52–1.47)	
Online news portal	1.23 (0.86–2.01)		0.68 (0.42–1.15)	
Research articles/Journal websites	**1.31 (0.98**–**2.09)**		**1.03 (0.74**–**1.69)**	
TV news	0.86 (0.58–1.57)		0.94 (0.68–1.54)	

Males exhibited a significant association with both good knowledge (AOR 1.35, 95 % CI 0.92–2.14) and positive attitudes (AOR 1.18, 95 % CI 0.78–2.07). Additionally, individuals with Doctorate degrees had 1.67 times higher odds of good knowledge (95 % CI 1.21–2.49) and 1.53 times higher positive attitudes (AOR 1.53, 95 % CI 1.12–2.34) compared to Master's degree holders. Healthcare workers with less than 1 year of experience also showed significant associations with good knowledge (AOR 1.28, 95 % CI 0.92–2.09) and positive attitudes (AOR 1.42, 95 % CI 0.98–2.34). Furthermore, full-time employees (AOR 1.45, 95 % CI 1.12–2.34) and those who attended AI-related conferences (AOR 1.47, 95 % CI 1.12–2.34) displayed significantly higher levels of knowledge and positive attitudes.

### 3.6 Correlation between knowledge and attitude

Table 5 presents the correlation between knowledge and attitude concerning the adoption of artificial intelligence in healthcare. The table includes two scales: Positive attitude and Negative attitude. For individuals with good knowledge, there is a strong positive correlation (r = 0.89, P < 0.001) with a positive attitude, indicating that higher levels of knowledge are associated with a more positive attitude toward artificial intelligence adoption in healthcare. Conversely, for those with poor knowledge, there is also a significant positive correlation (r = 0.65, P < 0.001) with a negative attitude, suggesting that lower levels of knowledge are linked to a less favorable attitude regarding the adoption of artificial intelligence in healthcare. The correlation values and statistical significance provide insights into the relationship between knowledge and attitudes, offering a quantitative perspective on how these variables are interconnected in the context of artificial intelligence adoption in the healthcare sector.

**Table 5:** Illustrates the correlation between knowledge and attitude regarding the adoption of artificial intelligence in healthcare.

Scales	Positive attitude	Negative attitude
Good knowledge	**r = 0.89 (P < 0.001)**	
Poor knowledge		**r = 0.65 (P < 0.001)**

### 3.7 Prevalence of knowledge and attitude

**Fig. 2:**
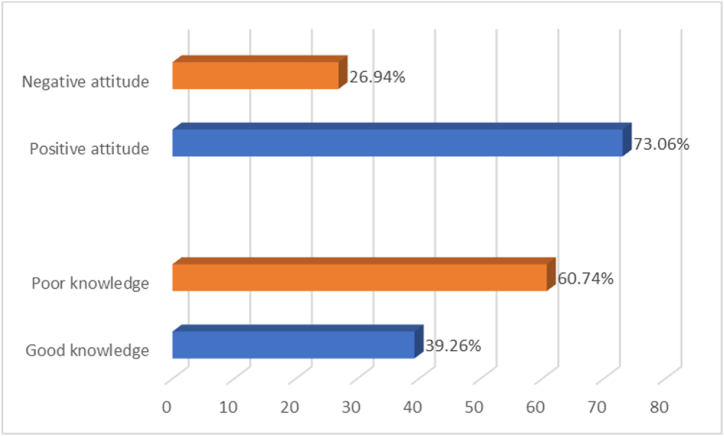
Prevalence of knowledge and attitude.

Fig. 2 presents the prevalence of knowledge and attitude regarding the adoption of artificial intelligence in healthcare, expressed in percentages. In terms of knowledge, 39.26 % of the participants are categorized as having good knowledge, while 60.74 % fall into the category of poor knowledge. This indicates that a majority of the respondents had a lower level of knowledge regarding artificial intelligence in healthcare. Regarding attitude, Fig. 2 indicates that 73.06 % of the participants exhibited a positive attitude toward the adoption of artificial intelligence in healthcare, while 26.94 % had a negative attitude. This suggests that a significant portion of the surveyed individuals hold a positive perspective on the incorporation of artificial intelligence in the healthcare domain. The prevalence percentages provide an overview of the distribution of knowledge and attitudes among the participants, offering insights into the general trends within the surveyed population.

### 3.8 Factors influencing AI knowledge and attitudes

**Fig. 3:**
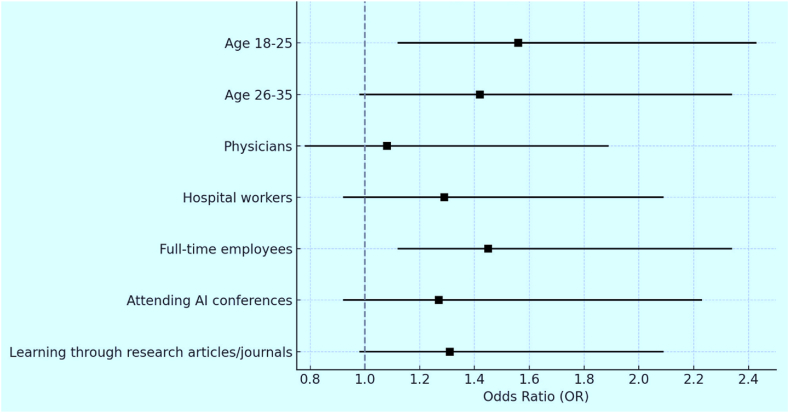
Forest plot of factors influencing AI knowledge and attitudes.

The forest plot presents (see Fig. 3) the adjusted odds ratios (AOR) and 95 % confidence intervals (CI) for factors influencing healthcare workers' knowledge and attitudes towards AI adoption. The plot highlights significant associations for the age group 18–25 (AOR: 1.56, 95 % CI: 1.12–2.43) and full-time employees (AOR: 1.45, 95 % CI: 1.12–2.34), indicating these groups are more likely to possess good AI knowledge and positive attitudes. Other factors, including age group 26–35, physicians, hospital workers, and educational exposure to AI, show positive trends, though their CIs cross 1, suggesting less certainty. The summary measure illustrates the overall effect, reinforcing the importance of age and employment status as key predictors.

## 4 Discussion

This study holds significant importance as it sheds light on the knowledge and attitudes of healthcare workers toward the adoption of artificial intelligence (AI), a critical area for modern healthcare systems. In an era where AI is poised to transform clinical practices, understanding these factors is essential for its successful integration [45]. Our findings highlight a crucial gap in healthcare workers' knowledge about AI, with a considerable proportion of participants displaying low awareness. This is a concerning observation given the growing role of AI in healthcare [46]. Knowledge plays a foundational role in fostering confidence and competence in utilizing new technologies, and the lack of knowledge could hinder the effective implementation of AI in clinical environments [47]. Thus, this study underscores the need for tailored educational interventions aimed at enhancing healthcare professionals' understanding of AI. Moreover, the integration of AI raises concerns around patient data privacy, as AI systems rely heavily on large datasets, which often contain sensitive patient information [48]. Ensuring data security and compliance with privacy regulations is essential to maintain trust and protect patient rights [49].

Several factors were associated with healthcare workers' knowledge of AI. Notably, those with more exposure to AI technologies, either through direct use or professional development courses, were more likely to possess higher levels of knowledge. This finding aligns with similar studies that emphasize the importance of ongoing training and learning opportunities in keeping healthcare professionals updated on technological advances. For example, Hamedani et al. (2023) found that structured educational programs significantly improved healthcare workers’ knowledge of AI, reinforcing the idea that targeted training initiatives are key to bridging the knowledge gap [47].

Our study also explored the attitudes of healthcare workers toward AI adoption, revealing generally positive perceptions. Most participants expressed a willingness to incorporate AI into their daily routines, recognizing its potential to enhance patient care and operational efficiency. This openness is an encouraging sign for the future of AI in healthcare, as positive attitudes are pivotal for seamless technology integration [50]. Similar findings by Swed et al. (2022) showed that healthcare workers with positive attitudes toward AI were more likely to engage in continuous learning about emerging technologies [51]. These studies collectively highlight the interplay between knowledge, attitudes, and the likelihood of successful AI adoption.

However, it is important to note that not all attitudes were uniformly positive. Some healthcare professionals expressed concerns about job displacement, ethical challenges, and the reliability of AI-driven decisions, which could hinder AI's widespread acceptance. The potential for AI to automate certain clinical tasks raises fears among healthcare professionals about job security, particularly in roles that may be more easily replaced by technology [52]. Addressing these concerns is essential for developing a workforce that feels secure and valued in the face of AI advancements [53]. Such apprehensions are consistent with broader societal concerns about AI, suggesting that addressing these issues is critical for fostering a more favorable environment for its adoption [54]. Our study underscores the need for healthcare systems to not only focus on improving knowledge but also on addressing the socio-ethical implications of AI, such as algorithmic bias, patient data privacy, and the impact on professional roles.

In terms of the causes of the associations observed, the study suggests that knowledge and attitudes are interconnected. Higher levels of knowledge were often linked to more positive attitudes toward AI, likely due to increased familiarity and comfort with the technology. This is supported by research indicating that individuals with greater technological literacy are more likely to accept new innovations [55]. However, it is crucial to recognize that increased knowledge alone may not lead to uniform acceptance [56]. Concerns about AI's ethical implications and its potential to disrupt existing professional roles need to be addressed to ensure a balanced perspective on its adoption [57].

This study highlights important shifts in the healthcare workforce's readiness for AI. While knowledge gaps remain a significant barrier, the generally positive attitudes toward AI provide a strong foundation for its future integration. To ensure successful adoption, a multifaceted approach is needed—one that enhances healthcare professionals' knowledge, safeguards patient data privacy, addresses ethical concerns, and fosters continuous learning and professional development [58]. By doing so, healthcare systems can create a collaborative environment where AI technologies can be effectively integrated, ultimately leading to improved patient outcomes and greater healthcare efficiency [59].

### 4.1 Implications of this study

The findings of this research underscore the pivotal role of frontline healthcare professionals as key stakeholders in the ongoing AI revolution within the medical domain. As we navigate the complex terrain of AI adoption, understanding the knowledge base and attitudes of healthcare workers becomes instrumental in fostering a collaborative and receptive environment. This research acts as a mirror, reflecting the intricate dance between human intuition and artificial intelligence. The nuanced perspectives unveiled provide strategic insights for policymakers, educators, and technology developers alike, guiding the formulation of training programs, ethical frameworks, and user-friendly AI interfaces tailored to the specific needs of healthcare workers. Moreover, these insights hold the potential to bridge the gap between technological innovation and human-centric care, ensuring that AI is not perceived as a threat but rather as a valuable ally in augmenting healthcare delivery.

## 5 Strengths and limitations

The strengths of this study lie in its use of a cross-sectional design, which allowed for the collection of real-time data on healthcare professionals’ perspectives on artificial intelligence (AI) adoption. The study employed a stratified random sampling technique to ensure a diverse representation of participants across different healthcare settings, enhancing the generalizability of the results. Additionally, the inclusion of healthcare professionals with at least six months of direct patient care experience, along with relevant educational qualifications, ensured the relevance and depth of the insights gathered. The survey instrument, validated through pilot testing, further strengthened the reliability and accuracy of the collected data.

However, the study has some limitations. The use of self-administered surveys, both in electronic and paper formats, may introduce response bias, as participants could provide socially desirable answers. The sampling method, though diverse, may not capture all healthcare settings in Dhaka, and convenience sampling could introduce selection bias. The reliance on self-reported data, especially concerning knowledge and attitudes toward AI, may also lead to recall bias or differences in participants' interpretations of AI concepts. Furthermore, the study is limited to Dhaka, Bangladesh, which may restrict the applicability of the findings to other regions or countries with different healthcare systems and varying healthcare infrastructures.

## 6 Recommendations for future research

Building on the findings of this study, several specific recommendations for future research emerge. First, more targeted research is needed to explore the impact of educational interventions on improving healthcare professionals' knowledge of AI. Given the strong association between knowledge and positive attitudes identified in this study, designing and evaluating AI-focused training programs across different healthcare settings could provide insights into the most effective strategies for knowledge enhancement. Such studies could assess how tailored educational curricula affect both immediate knowledge retention and long-term attitudes toward AI integration in clinical practice. Second, future research should investigate the role of demographic factors, such as age and professional role, which this study found to significantly influence both knowledge and attitudes. Exploring why younger healthcare professionals and certain job roles, like physicians, are more knowledgeable and open to AI adoption could reveal key motivators and barriers. This could help in designing targeted interventions that address specific knowledge gaps or concerns based on demographics.

Additionally, qualitative research methods, such as focus groups or in-depth interviews, could be employed to understand the underlying reasons behind healthcare workers’ apprehensions regarding AI, such as concerns over job displacement or ethical implications. This would provide a deeper understanding of the social and psychological factors influencing attitudes and allow for more nuanced recommendations on how to address these concerns. Finally, future studies should explore the long-term impact of AI adoption on healthcare delivery, particularly in patient outcomes and healthcare efficiency. Longitudinal studies following the implementation of AI tools in various clinical settings would be valuable to determine whether the positive attitudes observed translate into tangible improvements in care and operational processes. This will help validate the practical benefits of AI integration, beyond theoretical knowledge and attitudes.

## 7 Conclusions

The findings underscore the critical need for targeted educational interventions to bridge knowledge gaps among healthcare professionals. As the healthcare industry increasingly integrates artificial intelligence (AI) technologies, grasping and embracing these innovations become paramount for delivering optimal patient care. It is evident that a positive correlation exists between healthcare workers' familiarity with AI and their favorable attitudes towards its adoption. Institutions should prioritize comprehensive training programs that demystify AI, emphasizing its potential to enhance diagnostic accuracy, streamline administrative tasks, and ultimately improve patient outcomes. Furthermore, initiatives promoting an open dialogue on ethical considerations surrounding AI in healthcare must be championed. While the study identifies pockets of skepticism among healthcare workers, it also highlights the potential for a transformative shift in attitudes through targeted education and exposure. Collaboration between AI developers and healthcare professionals is crucial for designing user-friendly interfaces and ensuring AI solutions align with the unique needs of healthcare settings. As the healthcare landscape evolves, fostering a culture of continuous learning and adaptability will be instrumental in maximizing the benefits of AI adoption while addressing the concerns of frontline healthcare workers.

## CRediT authorship contribution statement

**Moustaq Karim Khan Rony:** Writing – review & editing, Writing – original draft, Visualization, Validation, Supervision, Software, Resources, Project administration, Methodology, Investigation, Formal analysis, Data curation, Conceptualization. **Khadiza Akter:** Writing – review & editing, Writing – original draft, Software, Resources, Data curation, Conceptualization. **Latifun Nesa:** Writing – review & editing, Visualization, Resources, Project administration, Investigation, Data curation. **Md Tawhidul Islam:** Writing – original draft, Validation, Resources, Investigation, Data curation. **Fateha Tuj Johra:** Writing – review & editing, Supervision, Resources, Project administration, Formal analysis. **Fazila Akter:** Writing – review & editing, Validation, Software, Conceptualization. **Muhammad Join Uddin:** Writing – review & editing, Methodology, Investigation, Data curation. **Jeni Begum:** Writing – review & editing, Resources, Methodology, Data curation. **Md. Abdun Noor:** Writing – review & editing, Visualization, Data curation, Conceptualization. **Sumon Ahmad:** Writing – review & editing, Resources, Investigation, Formal analysis. **Sabren Mukta Tanha:** Writing – review & editing, Visualization, Supervision, Data curation. **Most. Tahmina Khatun:** Writing – review & editing, Resources, Conceptualization. **Shuvashish Das Bala:** Writing – review & editing, Supervision, Conceptualization. **Mst. Rina Parvin:** Writing – review & editing, Writing – original draft, Visualization, Supervision, Methodology, Formal analysis, Data curation, Conceptualization.

## Ethics statement

Prior to initiating the study, the investigators diligently secured approval from the ethics review board of Mahbubur Rahman Memorial Hospital & Nursing Institute (approval number: MRMHNI/IRB/HR-20230513) on May 5, 2023, affirming their commitment to ethical guidelines and the protection of the well-being of participating healthcare workers.

## Participants' informed consent

All participants provided informed consent, emphasizing the study's objectives, potential risks, and the voluntary nature of their involvement.

## Data and code availability statement

Data will be made available on request.

## Declaration of competing interest

The authors declare that they have no known competing financial interests or personal relationships that could have appeared to influence the work reported in this paper.
